# Draft Genome Sequence of *Amphirosellinia nigrospora* JS-1675, an Endophytic Fungus from *Pteris cretica*

**DOI:** 10.1128/MRA.00069-19

**Published:** 2019-05-16

**Authors:** Jongbum Jeon, Sook-Young Park, Jung A. Kim, Nan Hee Yu, Ae Ran Park, Jin-Cheol Kim, Yong-Hwan Lee, Soonok Kim

**Affiliations:** a Department of Agricultural Biotechnology, Interdisciplinary Program in Agricultural Genomics, Center for Fungal Genetic Resources, Seoul National University, Seoul, South Korea; b Department of Plant Medicine, Sunchon National University, Suncheon, South Korea; c Genetic Resources Assessment Division, National Institute of Biological Resources, Incheon, South Korea; d Department of Agricultural Chemistry, Institute of Environmentally Friendly Agriculture, Chonnam National University, Gwangju, South Korea; Vanderbilt University

## Abstract

The fungus Amphirosellinia nigrospora strain JS-1675 has been reported to exert antimicrobial effects against various plant-pathogenic bacteria and fungi. Here, we report the draft genome sequence of A. nigrospora for the first time. The assembly comprises 48,177,783 bp with 18 scaffolds.

## Abstract (precis)

The fungus Amphirosellinia nigrospora strain JS-1675 has been reported to exert antimicrobial effects against various plant-pathogenic bacteria and fungi. Here, we report the draft genome sequence of A. nigrospora for the first time.

## ANNOUNCEMENT

*Amphirosellinia nigrospora* (phylum Ascomycota, class Sordariomycetes, family Xylariaceae) strain JS-1675 is an endophytic fungus isolated from the Cretan brake fern (Pteris cretica). It has been reported to produce oxygenated cyclohexanone, a potent antimicrobial compound that is particularly effective against the bacterial wilt pathogen of tomato, Ralstonia solanacearum, as well as against various agronomically significant phytopathogenic fungi (1). It is generally accepted that the polyketide synthase (PKS), nonribosomal protein synthetase (NRPS), and terpene synthase (TS) genes are involved in the production of such secondary metabolites. The genome sequence of *A. nigrospora* strain JS-1675 will enable identification of genes involved in the production of oxygenated cyclohexanone.

Genomic DNA was extracted from fresh mycelia grown in potato dextrose broth at 25°C with shaking at 120 rpm for 2 days, using a DNeasy minikit (Qiagen, Valencia, CA, USA) according to the manufacturer’s instructions. Library preparation and genome sequencing were performed at Theragen Etex Bio Institute (Suwon, South Korea). A single-molecule real-time (SMRT) sequencing library was prepared according to the PacBio standard library preparation protocol with a fragment size of 20 kb. A total of 443,875 reads with an average size of 10.2 kb, and therefore 4.57 Gb of sequences, were generated on 4 cells of a PacBio RS II sequencer. These reads were assembled into 72 contigs with a total length of 48,704,170 bp and an *N*_50_ value of 4,754,034 bp using the overlap-layout-consensus (OLC) algorithm (2). Additionally, two short-read sequencing data sets were generated using 101 cycles of paired-end sequencing on an Illumina HiSeq 2000 platform from one short paired-end (PE) library (average insert size, 350 bp) and one long mate pair (MP) library (average insert size, 10 kb). A total of 45,165,534 reads with a Q30 of 79.38% were generated from the PE library. A total of 10,307,236 high-quality reads out of 75,404,568 reads were obtained from the MP library after filtering with NextClip v1.3 (3). These short reads were assembled using SOAPdenovo v2.04 to generate 1,182 contigs with a total length of 47,310,961 bp and an *N*_50_ value of 381,291 bp. The two assemblies were merged with HaploMerger2 (4) to 31 contigs, and scaffolding and gap filling were conducted sequentially using SSPACE-Standard v3.0 (5), SSPACE-LongRead v1.1 (6), and GapFiller v1.10 (7). Finally, from the 16.75 Gb (347.67× genome size) of genome sequences, the genome reads containing the nuclear genome were assembled into 18 scaffolds (31 contigs) with a total length of 48,177,783 bp and an *N*_50_ value of 11.78 Mb. The genome assembly was validated using BUSCO v. 3.0.2b, which showed an 80.3% hit against 290 fungal genes (8), and Core Eukaryotic Genes Mapping Approach (CEGMA) v.2.5, which showed a 93.95% hit against 248 core eukaryotic genes (9). The GC content of the assembled genome was 43.85%.

A total of 10,232 protein-coding genes were identified after gene prediction and annotation based on AUGUSTUS v.3.2.1 (10). After combining BLAST search results against UniProt or NCBI nonredundant (nr) and InterPro scan data, 9,745 genes were functionally annotated. Among these, 22 PKS and 10 NRPS biosynthetic gene clusters (BGCs) were identified through antiSMASH v4.0 (11, 12). Moreover, we found 307 transcription factor genes by the Fungal Transcription Factor Database (FTFD) v1.0 (13), 147 cytochrome P450 genes by the Fungal Cytochrome P450 Database v1.0 (14), 53 genes encoding plant cell wall-degrading enzymes by the Fungal Plant Cell Wall-Degrading Enzyme Database v1.0 (15), and 10 genes encoding laccases and 29 genes encoding peroxidases using the Fungal Peroxide Database (fPoxDB) v1.0 (16).

This is the first genome sequenced in the genusAmphirosellinia. This draft genome sequence will provide valuable information to identify genes for biosynthesis of antimicrobial compounds and will also facilitate comparative genomics with other publicly available fungal species, especially those of the family Xylariaceae, for evolutionary studies.

### Data availability.

The whole-genome sequence of *Amphirosellinia nigrospora* JS-1675 obtained in this study has been deposited at GenBank under the accession no. SCHM00000000. The version described in this article is the first version, SCHM01000000. SRA data of PacBio sequences and Illumina sequences were also deposited at GenBank under accession no. SRR8463317, SRR8463318, and SRR8463316.
